# Developmental Signatures of Microbiota-Derived Metabolites in the Mouse Brain

**DOI:** 10.3390/metabo10050172

**Published:** 2020-04-25

**Authors:** Jonathan R. Swann, Sonia O. Spitzer, Rochellys Diaz Heijtz

**Affiliations:** 1 School of Human Development and Health, Faculty of Medicine, University of Southampton, University Road, Southampton SO17 1BJ, UK; 2 Department of Metabolism, Digestion and Reproduction, Faculty of Medicine, Imperial College London, London SW7 2AZ, UK; 3 Department of Neuroscience, Karolinska Institute, 171 77 Stockholm, Sweden; 4 The Francis Crick Institute, London, 1 Midland Rd, London NW1 1AT, UK; 5 INSERM U1239, University of Rouen, Normandy, 76130 Mont-Saint-Aignan, France

**Keywords:** microbiota, microbiome, forebrain, metabolome, gut–brain axis, mass spectrometry, development, imidazole propionate, trimethylamine-*N*-oxide, indoxyl-sulfate, indolelactate, hippurate, phenol-sulfate, phenylacetylglutamine

## Abstract

The gut microbiome is recognized to exert a wide-ranging influence on host health and disease, including brain development and behavior. Commensal bacteria can produce bioactive molecules that enter the circulation and impact host physiology and homeostasis. However, little is known about the potential for these metabolites to cross the blood–brain barrier and enter the developing brain under normal physiological conditions. In this study, we used a liquid chromatography–mass spectrometry-based metabolomic approach to characterize the developmental profiles of microbial-derived metabolites in the forebrains of mice across three key postnatal developmental stages, co-occurring with the maturation of the gut microbiota. We demonstrate that direct metabolites of the gut microbiome (e.g., imidazole propionate) or products of the combinatorial metabolism between the microbiome and host (e.g., 3-indoxyl-sulfate, trimethylamine-*N*-oxide, and phenylacetylglycine) are present in the forebrains of mice as early as the neonatal period and remain into adulthood. These findings demonstrate that microbial-associated molecules can cross the BBB either in their detected form or as precursor molecules that undergo further processing in the brain. These chemical messengers are able to bind receptors known to be expressed in the brain. Alterations in the gut microbiome may therefore influence neurodevelopmental trajectories via the regulation of these microbial-associated metabolites.

## 1. Introduction

During birth and rapidly thereafter, the mammalian gastrointestinal (GI) tract is colonized by trillions of microorganisms, including bacteria, archaea, fungi, and viruses—known collectively as the gut microbiota. The bacterial component of this microbial ecosystem is currently the most characterized. The combined genetic material of all bacteria in the gut exceeds the human genome by at least a factor of 100 [1,2]. Many of these bacterial genes encode enzymes that can perform metabolic functions not achievable by the host and produce metabolites that can impact host health and disease. It is now widely recognized that gut bacteria interact with and perform multiple critical functions for their host, including dietary extraction, the production of vitamins, and providing protection against invading pathogens [3,4]. During the last few decades, studies have revealed that gut microbiota exert a broader range of effects on host physiology and development beyond the GI tract, including the modulation of brain development and behavior [5,6]. However, the precise molecular mechanisms mediating interactions between gut microbes and the developing brain remain to be clearly defined.

Commensal gut bacteria can transform dietary components, such as macronutrients and micronutrients, into a wide range of metabolites, which can impact host physiology and homeostasis. These metabolites include short-chain fatty acids (e.g., acetate, propionate, and butyrate; end products of the fermentation of dietary fibers), methylamines (derived from food containing lecithin, choline, and *L*-carnitine), amino acid derivatives, and vitamins, which are an integral part of the host metabolome. Moreover, the composition of the gut microbiota has a large impact on the metabolic profile of the plasma. Using an untargeted mass spectrometry-based approach to compare plasma from germ-free (GF; devoid of bacteria through life) and conventional mice, Wikoff and colleagues demonstrated that several biochemical pathways were disrupted in the absence of microbiota [7]. This included the metabolic processing of indole-containing metabolites derived from tryptophan, such as the antioxidant indole-3-propionic acid. Recently, it was found that an overproduction of indole in the gut was associated with greater anxiety-like and despair behavior in rodents [8]. Moreover, a role of host-microbe tryptophan metabolism has been increasingly recognized as an important signaling pathway along the microbiota–gut–brain axis [9]. However, little is known regarding the developmental profile of microbial-derived metabolites in the healthy developing brain and/or their potential role in neurodevelopment. In this study, we used a liquid chromatography-mass spectrometry (LCMS)-based metabolomic approach to broadly characterize the neurobiochemical profile of the forebrain of mice at three important developmental milestones co-occurring with the maturation of the gut microbiota. This included post-natal days 3, 21, and 60 reflecting the neonatal, prepubertal/juvenile, and young adult period, respectively. Special attention was paid towards metabolites related to gut microbial activity.

## 2. Results

The biochemical profiles of the forebrain were measured at all ages using a combination of liquid chromatography–mass spectrometry approaches covering a broad range of hydrophilic and hydrophobic compounds. This combination of approaches measured a total of 469 metabolites in the forebrain.

Principal components analysis (PCA) was performed on the forebrain profiles to identify sources of metabolic variation in the dataset. The scores plot generated from the PCA model comparing the neurobiochemical profiles of all ages shows clear separation by age (Supplementary Figure S1). The largest variation in the metabolic profiles (principal component 1 (PC1), 82.8% variance) was observed between post-natal day (P)3 and the two older age groups (P21 and P60). The main drivers behind this separation were the higher amounts of proline, taurine, phosphocholine, adenosine monophosphate, citrulline, 1-palmitoyl-2-palmitoleoyl-glycerophosphorylcholine, trans-4-hydroxyproline, threonine, and spermidine in the P3 animals compared to in the older mice and the lower amounts of glutamate, creatine, adenosine, *N*-acetylaspartate, glutamine, γ-aminobutyric acid (GABA), stearate, stearoyl sphingomyelin, arachidonate, glycerophosphorylcholine, oleate, palmitate, and docosahexaenoate. Separation was seen along PC2 (6% variance) between the forebrain profiles of P21 and P60 mice. The prepubertal (P21) forebrain had greater amounts of *N*-acetylaspartate, glutamine, glutamate, GABA, creatine, and stearoyl sphingomyelin, while the young adult (P60) forebrain contained higher adenosine, glycerophosphorylcholine, the polyunsaturated fatty acids docosahexaenoic acid (ω-3) and arachidonic acid (ω-6), the monounsaturated fatty acid oleic acid, and the saturated fatty acids, palmitic acid and stearic acid.

**Figure 1:**
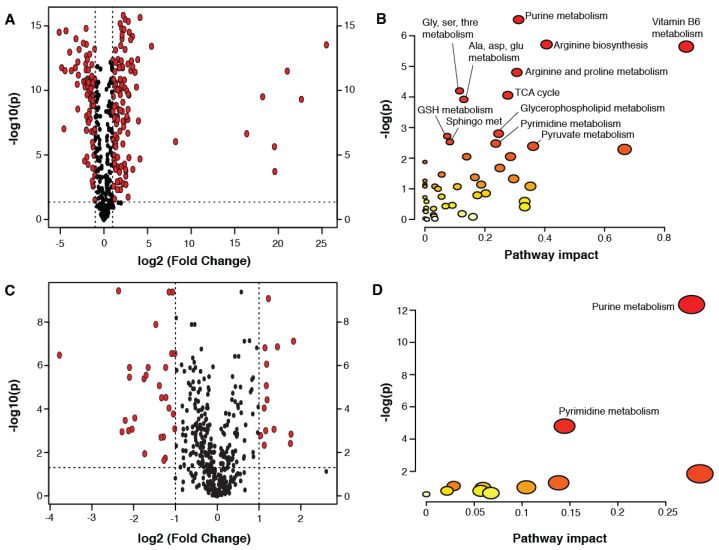
Pairwise comparisons between the metabolites measured in the forebrain at P3, 21, and 60. (**A**) Volcano plot comparing the forebrain metabolites at P21 versus P3; (**B**) Pathway analysis performed on the metabolites found to be significantly different between P21 and P3; (**C**) Volcano plot comparing the forebrain metabolites at P60 versus P21; (**D**) Pathway analysis performed on the metabolites found to be significantly different between P60 and P21. Significant metabolites were >2 fold different between the age groups compared with an FDR adjusted *p* < 0.05. Pathway impact values derived from pathway topology analysis (relative-betweenness centrality). Significantly enriched pathways are labeled (*p* < 0.1). Node radius is defined by its pathway impact value and the node color is determined by its *p* value.

For a detailed inspection of the age-related changes occurring in the forebrain, pairwise comparisons were made between the groups (thresholds for significant metabolites: fold change >2; FDR adjusted *p* < 0.05; the results are presented in volcano plots). A total of 121 metabolites were higher in the P21 forebrain compared to the P3 forebrain while 78 metabolites were lower (Figure 1A; significant metabolites listed in Supplementary Table S1). Several metabolites derived from the gut microbiota were observed to be higher in the P21 forebrain compared to that of P3 animals. This included imidazole propionate, 3-indoxyl sulfate (3-IS), and phenol sulfate. The gut microbial metabolite, indolelactate, was lower in the P21 brain compared to in the P3 brain. Other metabolites that can be both microbial and host in origin also differed with age, including lactate (increased), pipecolic acid (decreased), and the polyamines putrescine (decreased) and spermidine (decreased). Other notable changes included several eicosanoid-related metabolites, metabolites involved in sphingolipid metabolism, endocannabinoids (*N*-stearoyltaurine and oleoylethanolamide), and the neurotransmitters acetylcholine and *N*-acetylaspartate. Pathway analysis was performed on these significant age-related metabolites. This identified purine, pyrimidine, vitamin B6, arginine, proline, glycine, serine, threonine, alanine, aspartate, glutamate, glutathione, sphingolipid, and glycerophosphorylcholine metabolism to be significantly different in the forebrain between these ages, as well as the TCA cycle, pyruvate metabolism, and arginine biosynthesis (Figure 1B).

Fewer metabolic differences were observed in the forebrain between P21 and P60 mice (43 metabolites) compared to those occurring between P3 and P21. A total of 14 metabolites were more abundant in the forebrain at P60 compared to at P21, while 29 metabolites were less abundant (Figure 1C; significant metabolite changes listed in Supplementary Table S2). Pathway analysis revealed significant differences in purine and pyrimidine metabolism between these ages. Metabolites involved in nicotinamide metabolism (1-methylnicotinamide and *N*-methylnicotinic acid (trigonelline)) were observed to be lower in the forebrain of P60 mice compared to P21 mice.

**Figure 2:**
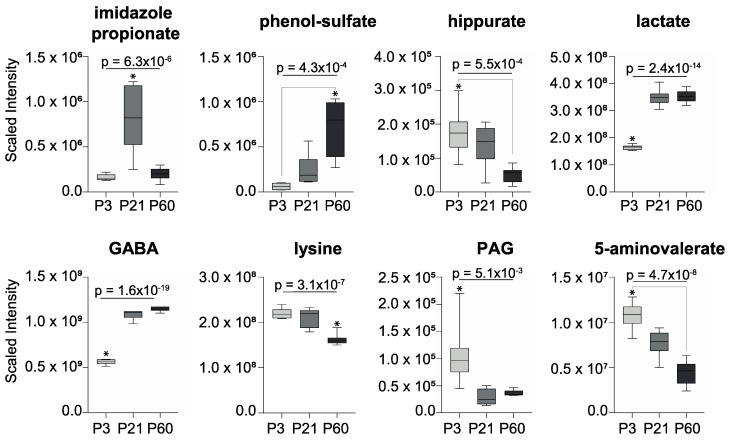
Gut microbial-related metabolites measured in the forebrain that vary with age (* *p* < 0.05; one-way ANOVA with Bonferroni post-hoc tests). γ-aminobutyric acid, GABA; phenylacetylglycine, PAG.

Several metabolites related to gut microbial metabolism were measured in the forebrain and observed to change significantly with age (Figure 2; *p* < 0.05, one-way ANOVA with Bonferroni post-hoc test). Lactate, γ-aminobutyric acid (GABA), and phenol sulfate progressively increased from P3 to P60 while hippurate, lysine, and 5-aminovalerate decreased across these ages. Imidazole propionate increased from P3 to P21 before decreasing by P60. Conversely, phenylacetylglycine (PAG) decreased from P3 to P21 before slightly increasing at P60. It should be noted that GABA, lactate, lysine, and 5-aminovalerate can arise from both host endogenous metabolism and that of the gut microbiota.

**Figure 3:**
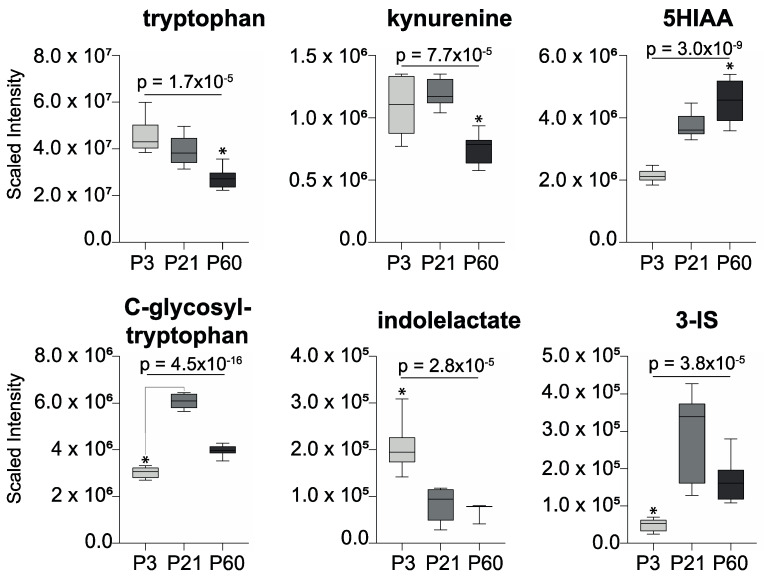
Tryptophan-related metabolites measured in the forebrain that vary with age (* *p* < 0.05; one-way ANOVA with Bonferroni post-hoc tests). 3-indoxyl-sulfate, 3-IS; 5-hydroxy-indoleacetate, 5-HIAA.

Tryptophan and tryptophan-related metabolites that are produced by the mammalian host and the intestinal microbiota were also found to fluctuate in the forebrain across the ages (Figure 3). Tryptophan and kynurenine declined with age, while 5-hydroxyindole acetate (5HIAA), the main metabolite of serotonin, increased with age. *C*-glycosyl-tryptophan (also known as *C*-mannosyl-tryptophan) increased in the forebrain from P3 to P21 and then decreased by P60. Indolelactate, which can be produced by the gut microbiota, was observed to decrease in the forebrain across this age range, while 3-indoxyl-sulfate, a product of gut microbial–host co-metabolism, peaked in abundance at P21 and then declined by P60.

**Figure 4:**
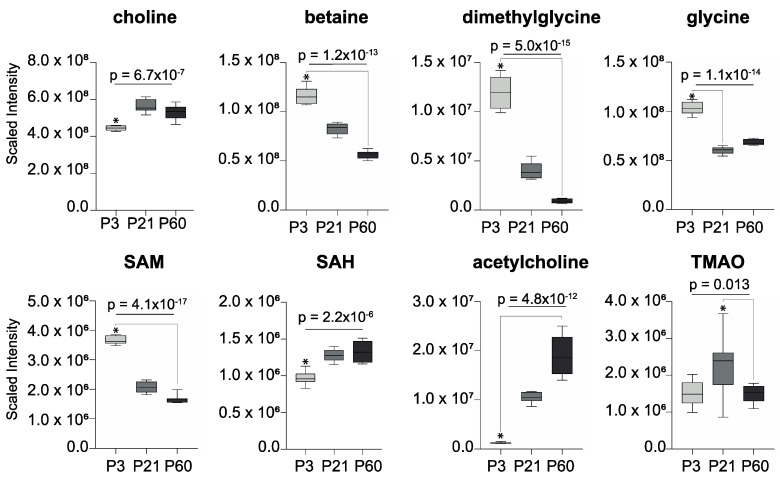
Choline-related metabolites measured in the forebrain that vary with age (* *p* < 0.05; one-way ANOVA with Bonferroni post-hoc tests). *S*-adenosyl-homocysteine, SAH; *S*-adenosyl-methionine, SAM; trimethylamine-*N*-oxide, TMAO.

Several choline-related metabolites were noted to change with age (Figure 4). Betaine, dimethylglycine, and *S*-adenosyl-methionine (SAM) were higher in the forebrain at P3 before becoming progressively less abundant with age. *S*-adenosyl-homocysteine (SAH) followed the opposite trend to its precursor SAM, becoming significantly more abundant with age, as did the neurotransmitter acetylcholine. Choline was least abundant in the forebrain at P3 and most abundant at P21. Trimethylamine-*N*-oxide (TMAO), a microbial–host co-metabolite of choline, was detected in the forebrain and followed the same trend as choline, being most abundant at P21.

## 3. Discussion

Clear changes were seen in the biomolecular landscape of the forebrain as it progressed from the neonatal to the pre-pubescent phase and then into young adulthood. These processes were biochemically diverse and indicate the dynamic shifts in the metabolic demands of the developing brain. Changes in energy metabolism (pyruvate metabolism, the TCA cycle, and fatty acid oxidation) were accompanied by alterations in metabolites associated with neurotransmission (acetylcholine, glutamate, GABA, and *N*-acetylaspartate) and cholesterol, sphingolipid, and glycerophospholipid metabolism, as well as purine and pyrimidine metabolism. These metabolic changes reflect structural and functional modifications and are consistent with the overproduction of synapses and neuronal processes in early life before a period of activity-driven pruning in late childhood and adolescence [10].

The gut microbiota is recognized as a key player in the gut–brain axis with the potential to influence emotional behavior, cognitive function, and neurological disorders. We have previously demonstrated that the gut microbiota can influence brain physiology, such as the regulation of neurotransmission and synaptogenesis [11]. We have also shown that the microbiota can modulate the metabolic profiles of the prefrontal cortex and hippocampus of rodents [12] and that gut-derived acetate can migrate to the brain where it can impact on feeding behaviors [13]. In this study, we demonstrate that several metabolites derived from the gut microbiome can translocate from the gut into the forebrain of mice. These microbial-associated signals are present as early as the neonatal period and remain into adulthood. While some metabolites such as GABA, lactate, indolelactate, phenol-sulfate, polyamines (spermidine and putrescine), lysine, and its degradation product 5-aminovalerate can be produced either endogenously by the host or exogenously by the gut microbiota, other metabolites are direct products of the gut microbiome (e.g., imidazole-propionate) or derivatives of the combinatorial metabolism between the microbiome and genome. These latter microbial–host co-metabolites include 3-indoxyl-sulfate (3-IS), trimethylamine-*N*-oxide (TMAO), hippurate, and phenylacetylglycine (PAG).

These findings highlight that gut microbial-derived molecules are able to cross the blood–brain barrier (BBB) either in their detected form or as precursor molecules that undergo further host processing in the brain. Many of these metabolites act as ligands for host receptors that are present in the brain. For example, imidazole propionate arises from the bacterial metabolism of dietary histidine via urocanate and has been shown to signal to the host by activating mechanistic target of rapamycin complex 1 (mTORC1) [14]. MTORC1 is expressed in the brain and has crucial roles in neurogenesis and synaptic formation [15], and the inhibition of MTORC1 can block long-term memory formation [16]. Interestingly, imidazole propionate was observed to be lower in the serum of a maternal immune activation model of autistic spectrum disorders [17].

Tryptophan metabolism is a key pathway in the gut–brain axis known to be influenced by the gut microbiota. Tryptophan is an essential amino acid obtained from the diet and is the precursor for the neurotransmitter serotonin. It can be metabolized by many Gram-positive and Gram-negative bacteria to indole, which can be absorbed and further processed by the host to indoxyl, which is then sulfated to form 3-IS [18]. Tryptophan can be metabolized to various other catabolites including indolelactate. Previous studies have found *Lactobacilli* spp., *Bifidobacterium* spp., *Clostridium sporogenes*, and *Clostridium bartettii* to produce this molecule [19]. Both 3-IS and indolelactate were measured in the forebrain of the mice, with indolelactate being most abundant during the neonatal period before significantly reducing with age, and 3-IS was present in low amounts in the neonatal period, peaking during the prepubertal period before declining in young adulthood. During early life, the abundance of *Bifidobacterium* species (producers of indolelactate) is known to be high in breast-fed infants, which may contribute to the greater amounts of indolelactate in this period [20]. Given the diverse bacterial groups able to produce indole, the diversification of the microbiota following weaning may explain the increase in 3-IS at P21. However, microbial profiling would be required to confirm these hypotheses. These compounds are important signaling molecules involved in microbe–host interactions. 3-IS is virtually absent from the cerebrospinal fluid of GF mice and mice treated with antibiotics but can be restored by reconstituting the gut microbiota [21]. High amounts of 3-IS in the serum and urine are associated with cognitive impairment and severe depressive disorder, respectively [22,23]. Both 3-IS and indolelactate are ligands for the host aryl hydrocarbon receptor (AhR), a transcription factor expressed throughout the brain including on neurons, astrocytes, and endothelial cells forming the BBB [24]. The activation of this transcription factor alters the innate and adaptive immune responses, and 3-IS has been shown to activate AhR signaling in astrocytes to limit CNS inflammation [25,26]. Consistently, lower circulating amounts of this compound were measured in individuals with multiple sclerosis [26]. 3-IS also acts as a co-factor for the neuronal growth factor (NGF), inducing neurite outgrowth and differentiation in vitro by activating AhR [27]. In vivo, AhR participates in hippocampal neurogenesis, and AhR-deficient mice displayed impaired hippocampal-dependent contextual fear memory [28]. The potential for these microbial-derived AhR ligands to impact on brain development and function warrants further investigation.

The vast majority of the body’s tryptophan is converted to kynurenine by indoleamine 2,3-dioxygenase (IDO) and tryptophan-2,3-dioxygenase [29]. Kynurenine is highly susceptible to changes in the gut microbiota. Kynurenic metabolites increased with *Bifidobacterium infantis* supplementation and decreased with *Lactobacillus johnsonii* supplementation [30,31]. One mechanism through which the gut microbiota can alter kynurenic metabolites is through lipopolysaccharide (LPS), a Gram-negative bacterial cell wall component. LPS is a potent inflammogen that can translocate from the gut environment into the systemic circulation and trigger inflammation in the host. A controlled inflammatory response is important for protection against infection, but uncontrolled chronic inflammation can lead to tissue damage. To regulate this immune response, IDO is induced in the host following exposure to LPS. The IDO-induced breakdown of tryptophan increases the production of kynurenine, which can interact with AhR to promote regulatory T cells, facilitating tolerance to LPS, and assisting in the resolution of inflammation and tissue homeostasis. The induction of IDO by LPS can result in the “tryptophan steal”, increasing the conversion of tryptophan to kynurenine, reducing its availability for serotonin production. Through this mechanism, LPS has been shown to induce depressive-like behaviors in mice [32]. In this study, tryptophan steadily decreased in abundance in the forebrain from the neonatal period to young adulthood. Kynurenine was abundant during the neonatal period and increased modestly before declining in young adulthood. This modest increase in kynurenine at P21 may be consistent with increased exposure to LPS as the microbiota establishes, although this did not appear to influence the abundance of 5-hydroxyindoleacetate, the principal metabolite of serotonin.

Choline metabolism is another biochemical process through which the gut microbiome can participate in the gut–brain axis. Choline is largely derived from the diet and is the precursor for the neurotransmitter acetylcholine. The microbiota can influence the bioavailability of this precursor by metabolizing choline to trimethylamine, which, following absorption from the gut, is processed by the host to TMAO. Both choline and TMAO were detected in the brain, with both compounds exhibiting the same age-dependent patterns in abundance, peaking at day 21 before decreasing in adulthood. Acetylcholine followed a different trend, progressively increasing in the brain with age, suggesting that microbial choline metabolism may not significantly influence the abundance of this neurotransmitter in the mouse forebrain in a conventionally fed state. The importance of TMAO in the brain is currently unknown. It has been detected in the human cerebrospinal fluid and has been associated with Alzheimer’s disease as well as having neuroprotective properties [33,34,35]. It has also been implicated in the disruption of the BBB by reducing the expression of tight junction proteins [36]. TMAO can bind and activate protein kinase R-like endoplasmic reticulum kinase (PERK), a kinase that responds to cellular stress [37]. PERK has been attributed with a role in normal and pathological brain function, and reductions in the amount or activity of PERK in the hippocampi of young adults have been shown to enhance neuronal excitability and improve cognitive function [38].

Choline is also important for the generation of SAM, the body’s primary methylating agent. Choline is metabolized to betaine and then dimethylglycine, liberating a methyl group that drives the homocysteine–methionine pathway to synthesize SAM. This methyl donor is essential for DNA and histone methylation, which are key for brain development and function. This process is dependent on the availability of SAM, which is strongly influenced by the availability of choline and betaine. Previous work has demonstrated that the gut bacterial consumption of choline can reduce SAM bioavailability, resulting in a reduction in global DNA methylation patterns in the host and also in its offspring, with downstream consequences for anxiety-like behaviors [39]. Inhibiting such activity reversed these outcomes. In the present study, betaine, dimethylglycine, and SAM were most abundant in the neonatal forebrain, emphasizing the importance of these molecules in the brain during this critical window of development.

The weaning process is also likely to contribute to some of the metabolic perturbations observed. From approximately post-natal day 18, animals begin to gradually wean from the mother’s breast milk onto solid foods. This will change the macronutrients and micronutrients available for host metabolism and also the substrate available for the intestinal microbiota. It has been shown that the weaning process can alter a range of host pathways, including those showing age-related changes in the brain, such as choline, lipid, amino acid, and energy metabolism [40]. Weaning has also been found to modify the compositional and functional profile of the intestinal microbiota [41], and we have previously demonstrated that prolonged breast feeding (delayed weaning) alters the intestinal microbiota and the urinary metabolome, and increases depressive-like behavior in rats [42].

This study contributes to the growing body of research highlighting the influence of the gut microbiota on the biochemical processes occurring in the brain. Microbial-derived metabolites were identified in the brain, and several of these signals have been shown to bind and activate host receptors expressed in this organ. A limitation of this work was the absence of microbiological data. This information could enable bacterial species or genes in the gut to be associated with specific metabolites. These could then be validated in subsequent studies, and the potential to manipulate these signals through the modulation of their bacterial sources could be investigated. All of the bacterial-associated metabolites exhibit age-dependent patterns, but at present, little is known regarding the importance of their timing or the magnitude of the effects on brain development and function. Further work is necessary to dissect the downstream consequences of the premature or delayed appearance of these microbial signaling molecules and the extent of their bioactivity in the brain. All of the molecules identified are derived from the metabolic interactions between the gut microbiome and the diet. As such, it is tempting to speculate that early life adversity, such as malnutrition, high-fat diets, and antibiotic exposure, could impact neurodevelopmental trajectories and increase the risks for neurodevelopmental disorders by altering the amounts of these microbial-derived metabolites entering the brain.

## 4. Materials and Methods

### Animals and Sample Collection

Pregnant C57BL/6N female mice were obtained from Charles River Laboratories (Sulzfeld, Germany) and housed, individually, in standard plastic cages (Makrolon Type III, Tecniplast, Buguggiate, Italy) under controlled temperature, humidity, and light (12:12 h light–dark cycle) conditions. Food and water were available ad libitum. Brain tissues were collected from naive male C57BL/6N mice at various postnatal ages (postnatal days 3, 21, and 60) from multiple litters (*n* = 8 per group); the day of birth was defined as postnatal day (P)0. All animals were euthanized in the morning to avoid potential diurnal variation in the metabolic profiles. For the metabolic profiling, forebrains were rapidly dissected out on an ice-cold surface, wet weighed, frozen on dry ice, and stored at −80 °C until use. All experiments were conducted according to a protocol approved by the Ethics Committee on Animal Research, Stockholm North and in accordance with the European Communities Council Directive of 22 September 2010 (2010/63/EU).

### Liquid Chromatography–Mass Spectrometry-Based Metabolic Phenotyping

Metabolic profiling of the brain tissue was performed by Metabolon (Durham, NC, US). Samples were prepared using the automated MicroLab STAR^®^ system from Hamilton Company. Several recovery standards were added prior to the first step in the extraction process for QC purposes. Tissue was homogenized with methanol (Glen Mills GenoGrinder 2000) for 2 minutes, followed by centrifugation. The resulting extract was divided into four fractions: two for analysis by two separate reverse phase (RP)/UPLC-MS/MS methods with positive ion mode electrospray ionization (ESI), one for analysis by RP/UPLC-MS/MS with negative ion mode ESI, and one for analysis by HILIC/UPLC-MS/MS with negative ion mode ESI. All methods were performed using Waters ACQUITY ultra-performance liquid chromatography and a Thermo Scientific Q-Exactive high resolution/accurate mass spectrometer interfaced with a heated electrospray ionization (HESI-II) source and Orbitrap mass analyzer operating at 35,000 mass resolution.

The organic solvent was removed from the samples using a vacuum concentrator (TurboVap, Zymark). The dried sample was reconstituted in a solvent compatible with each of the four methods. One aliquot was analyzed using acidic positive ion conditions, chromatographically optimized for more hydrophilic compounds. Here, the extract was gradient eluted from a C18 column (Waters UPLC BEH C18-2.1 × 100 mm, 1.7 µm) using water and methanol containing 0.05% perfluoropentanoic acid (PFPA) and 0.1% formic acid (FA). Another fraction was also analyzed under acidic positive ion conditions using chromatography optimized for more hydrophobic compounds. This extract was gradient eluted using the same column with methanol, acetonitrile, water, 0.05% PFPA, and 0.01% FA and operated at an overall higher organic content. Another fraction was analyzed using basic negative ion optimized conditions using a separate dedicated C18 column. The basic extracts were gradient eluted from the column using methanol and water, with 6.5 mM ammonium bicarbonate at pH 8. The final fraction was analyzed using negative ionization following elution from a HILIC column (Waters UPLC BEH amide 2.1 × 150 mm, 1.7 µm) with a gradient consisting of water and acetonitrile with 10 mM ammonium formate at pH 10.8. The MS analysis alternated between MS and data-dependent MS^n^ scans using dynamic exclusion. The scan range covered 70–1000 m/z, varying slightly between methods.

### Data Analysis

Raw data were extracted, peak identified, and QC processed using the hardware and software of Metabolon. Compounds were identified by comparison to library entries of purified standards. Peaks were quantified according to area-under-the-curve. Metabolic data was imported into MetaboAnalyst (www.metaboanalyst.ca). Volcano plots were generated for pair-wise comparisons (P21 vs. P3/P60 vs. P21) using a two-fold change and an FDR-adjusted *p* < 0.05 threshold. Metabolites found to meet these criteria were used for pathway analysis, applying the hypergeometric test (over representation analysis; *p* < 0.05) and relative-betweenness centrality (pathway topology analysis), with the KEGG *Mus musculus* pathway library. Principal component analysis (PCA) was performed using mean centered and pareto scaled data. Analysis of individual metabolites was performed using SPSS Statistics 26 (IBM). Metabolite abundances were compared between time points (P3, P21, and P60) by one-way ANOVA and equality of means measured by the Brown–Forsythe test. All results were confirmed by Kruskal–Wallis one-way ANOVA. Differences between each age group (P3 vs. P21/P3 vs. adult/P21 vs. adult) were assessed using post hoc pairwise comparisons with Bonferroni correction for multiple tests. The box plot hinges represent the 25th and 75th percentiles, and the median is plotted as a midline. The whiskers represent the minimum and maximum values. The significance level alpha was defined as a *p* value < 0.05.

## Supplementary Materials

The following are available online at https://www.mdpi.com/2218-1989/10/5/172/s1, Figure S1: Table S1: Table S2.
