# Supplementary material for: Developmental Signatures of Microbiota-Derived Metabolites in the Mouse Brain
Source: Metabolites. 2020 Apr 25;10(5):172. doi: 10.3390/metabo10050172 (PMC7281085; doi:10.3390/metabo10050172)
Supplement: Supplementary file 1 [file metabolites-10-00172-s001.pdf]

Developmental signatures of microbiota-derived metabolites in the mouse brain

Jonathan Swann [1,2,3]*, Sonia Spitzer [4] and Rochellys Diaz-Heijtz [3,*]

Supplementary Information

Supplementary Figure 1: Principal components analysis (PCA) model constructed on the forebrain metabolic profiles of all study animals. A) Scores plot (PC1 v PC2) and B) loadings plot from the PCA model (R^2 = 95%) using mean centered and pareto scaled data. AMP, adenosine monophosphate; GABA, γ-aminobutyric acid; GPC, glycerophosphocholine.

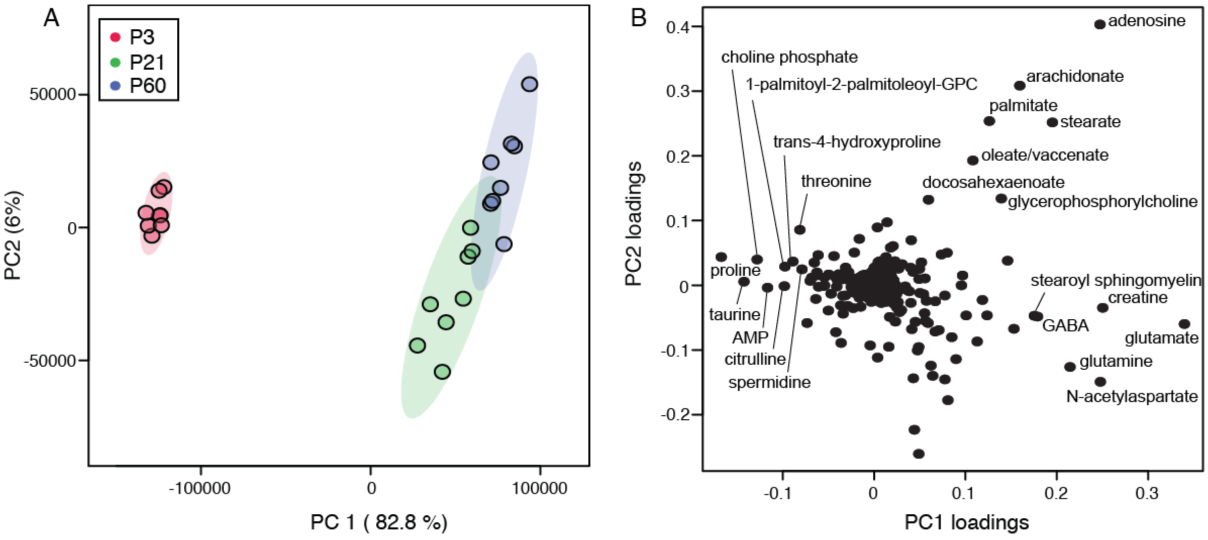

Supplementary Table S1: Forebrain metabolites significantly different between prepubertal (P21) and postnatal (P3). 121 metabolites were significantly higher in the forebrain of P21 mice compared to P3 mice and 78 metabolites significantly lower. Criteria for significance was greater than two fold-change (P21 relative to P3) and FDR-adjusted $P < 0.05$.

**Metabolite**	**Fold change (*P21 vs. P3*)**	**log2(FC)**	***p*.adj**	**-Log10(p)**
N-acetylaspartate	4.6362	2.2129	1.51E-16	15.822
pyroglutamine	17.699	4.1456	2.22E-16	15.653
1,2-dioleoyl-GPS 181/181	5.3136	2.4097	3.02E-16	15.52
sphingomyelin d181/181, d182/180	6.9231	2.7914	4.50E-16	15.347
1-stearoyl-2-arachidonoyl-GPC 180/204	3.6097	1.8519	6.67E-16	15.176
cystathionine	0.24157	-2.0495	1.57E-15	14.803
1-1-enyl-stearoyl-2-arachidonoyl-GPE P-180/204	4.0986	2.0351	1.57E-15	14.803
xanthine	4.956	2.3092	1.89E-15	14.724
putrescine	0.048905	-4.3539	2.41E-15	14.618
pro-hydroxy-pro	0.028596	-5.1281	3.10E-15	14.508
carnosine	7.4746	2.902	3.10E-15	14.508
sphingosine	4.2331	2.0817	5.05E-15	14.297
1-1-enyl-palmitoyl-2-palmitoyl-GPC P-160/160	9.6679	3.2732	6.53E-15	14.185
N-acetyltaurine	0.10845	-3.205	9.82E-15	14.008
adenine	4.2115	2.0743	9.82E-15	14.008
stearoyl sphingomyelin d181/180	8.3916	3.0689	1.17E-14	13.932
trans-4-hydroxyproline	0.19385	-2.367	2.17E-14	13.665
orotidine	0.24803	-2.0114	2.69E-14	13.571
homocarnosine	47585000	25.504	2.94E-14	13.532
1,2-dioleoyl-GPE 181/181	3.794	1.9237	3.36E-14	13.473
1-1-enyl-palmitoyl-2-oleoyl-GPC P-160/181	43.796	5.4527	3.80E-14	13.42
phosphoethanolamine	0.47563	-1.0721	6.13E-14	13.213
desmosterol	0.13929	-2.8438	6.75E-14	13.17
N-acetylglutamate	5.345	2.4182	6.75E-14	13.17
1-1-enyl-palmitoyl-2-arachidonoyl-GPE P-160/204	2.5172	1.3318	7.36E-14	13.133
7-methylguanine	0.06562	-3.9297	1.10E-13	12.96
3-hydroxy-3-methylglutarate	0.32287	-1.631	1.55E-13	12.811
2'-deoxycytidine	0.24939	-2.0035	2.11E-13	12.677
*N*6-carbamoylthreonyladenosine	0.26899	-1.8944	3.99E-13	12.399
1-1-enyl-stearoyl-2-oleoyl-GPE P-180/181	14.717	3.8794	4.07E-13	12.391
1-1-enyl-palmitoyl-2-linoleoyl-GPE P-160/182	3.0504	1.609	4.07E-13	12.391
1,2-dioleoyl-GPC 181/181	2.0284	1.0203	4.65E-13	12.333
sphingomyelin d181/200, d161/220	8.949	3.1617	5.12E-13	12.291
N-acetylarginine	3.9532	1.983	5.12E-13	12.291
citrate	3.9204	1.971	5.20E-13	12.284
maltotriose	0.12626	-2.9856	6.27E-13	12.202
N-acetylglutamine	2.5691	1.3612	6.65E-13	12.177
homoarginine	0.23484	-2.0902	8.69E-13	12.061
glycosyl-*N*-stearoyl-sphingosine	6.7299	2.7506	8.94E-13	12.049
dimethylarginine SDMA ADMA	0.38178	-1.3892	1.01E-12	11.997
sphingomyelin d181/221, d182/220, d161/241	4.3887	2.1338	1.51E-12	11.822
trigonelline	9.8812	3.3047	1.53E-12	11.816
4-imidazoleacetate	0.03481	-4.8443	1.76E-12	11.756
sphingomyelin d181/241, d182/240	3.4102	1.7699	1.76E-12	11.756
maltotetraose	0.10261	-3.2848	1.77E-12	11.752

N-acetylmethionine	4.3848	2.1325	1.77E-12	11.752
anserine	3.3	1.7225	1.77E-12	11.752
pseudouridine	0.29254	-1.7733	2.53E-12	11.597
1-palmitoleoyl-GPC 161	0.2421	-2.0463	2.59E-12	11.587
γ-glutamyl-ε-lysine	0.045241	-4.4662	2.94E-12	11.531
gulonic acid	0.075206	-3.733	2.95E-12	11.53
acetylcholine	8.4502	3.079	2.95E-12	11.53
3-sulfo-nervonoyl-galactosylceramide d181/241	2128800	21.022	3.18E-12	11.498
1-stearoyl-GPC 180	2.3743	1.2475	3.45E-12	11.462
sphingomyelin d181/170, d171/180, d191/160	11.889	3.5715	3.94E-12	11.404
guanidinoacetate	0.13129	-2.9292	6.50E-12	11.187
oxalate ethanedioate	0.3813	-1.391	8.01E-12	11.096
allantoin	0.27469	-1.8641	1.27E-11	10.897
N-acetylneuraminate	2.0588	1.0418	1.27E-11	10.897
N-palmitoyl-sphingosine d181/160	0.47795	-1.0651	1.29E-11	10.889
C-glycosyltryptophan	2.002	1.0015	1.32E-11	10.878
sphingomyelin d181/201, d182/200	3.6483	1.8672	1.37E-11	10.863
pyridoxal	2.1787	1.1235	1.49E-11	10.827
1-palmitoyl-2-palmitoleoyl-GPC 160/161	0.4379	-1.1913	1.54E-11	10.811
γ-glutamylglutamate	5.0684	2.3415	1.55E-11	10.809
cholesterol	3.62	1.856	1.59E-11	10.798
ornithine	0.23768	-2.0729	1.78E-11	10.749
succinate	0.27119	-1.8826	1.78E-11	10.749
inosine 5'-monophosphate	0.19888	-2.33	2.07E-11	10.683
UDP-glucuronate	0.29023	-1.7847	2.45E-11	10.611
2'-deoxyinosine	0.31248	-1.6782	2.57E-11	10.59
1-palmitoyl-2-stearoyl-GPC 160/180	2.4824	1.3117	2.57E-11	10.59
threonine	0.4181	-1.2581	2.59E-11	10.586
uridine 5'-monophosphate	0.11992	-3.0599	3.35E-11	10.475
1-palmitoleoyl-2-oleoyl-GPC 161/181	0.39027	-1.3575	3.77E-11	10.424
palmitoylcarnitine	4.1212	2.0431	3.82E-11	10.418
2-methylcitrate/homocitrate	2.519	1.3328	3.82E-11	10.418
cytidine 5'-monophosphate	0.4278	-1.225	3.91E-11	10.407
*N*2,*N*2-dimethylguanosine	0.27814	-1.8461	4.49E-11	10.348
cytidine 2',3'-cyclic monophosphate	0.34193	-1.5482	4.49E-11	10.348
ergothioneine	0.22421	-2.1571	5.42E-11	10.266
1-1-enyl-palmitoyl-2-arachidonoyl-GPC P-160/204	13.217	3.7243	5.57E-11	10.254
cysteine	4.3425	2.1185	5.91E-11	10.228
oleoylcarnitine	5.2648	2.3964	6.29E-11	10.201
Pipecolic acid	0.35955	-1.4758	7.38E-11	10.132
guanosine	3.3152	1.7291	7.87E-11	10.104
γ–glutamylglutamine	2.5648	1.3589	8.04E-11	10.095
pyruvate	2.9854	1.5779	9.35E-11	10.029
aconitate (cis or trans)	3.5558	1.8302	1.12E-10	9.9494
pyridoxamine phosphate	6.203	2.633	1.12E-10	9.9494
5-methyl-2'-deoxycytidine	0.30204	-1.7272	1.33E-10	9.8754
ribose 1-phosphate	6.9337	2.7936	2.03E-10	9.6927
stearoylcarnitine	3.6565	1.8704	2.11E-10	9.6757
N-palmitoyl-sphinganine d180/160	0.26183	-1.9333	2.19E-10	9.6598
2'-deoxycytidine 5'-monophosphate	0.34037	-1.5548	2.28E-10	9.6414
1-1-enyl-palmitoyl-2-oleoyl-GPE P-160/181	7.2675	2.8615	2.29E-10	9.6398
adenosine 5'-monophosphate	0.42299	-1.2413	2.40E-10	9.6196
lactate	2.1228	1.086	3.00E-10	9.5231
sphingomyelin d182/230, d181/231, d171/241	300280	18.196	3.11E-10	9.5072

1-palmitoyl-2-linoleoyl-GPE 160/182	0.38938	-1.3607	3.14E-10	9.5034
citrulline	0.39763	-1.3305	3.37E-10	9.472
β-hydroxyisovalerate	0.35465	-1.4955	3.95E-10	9.4037
saccharopine	6508800	22.634	4.90E-10	9.3099
glycerol 3-phosphate	0.34275	-1.5448	4.90E-10	9.3099
N-acetyltyrosine	0.41322	-1.275	6.15E-10	9.2111
pyridoxamine	4.5081	2.1725	8.22E-10	9.0849
guanosine 5'- monophosphate	0.44088	-1.1815	1.44E-09	8.8417
cysteinylglycine	2.6547	1.4085	1.45E-09	8.8399
3-methylcytidine	0.38138	-1.3907	1.50E-09	8.8234
behenoyl sphingomyelin d181/220	2.2784	1.188	1.82E-09	8.7409
uridine 5'-diphosphate	0.12475	-3.0029	2.80E-09	8.5532
adenosine	6.8208	2.7699	2.99E-09	8.5238
malonylcarnitine	3.3321	1.7364	3.21E-09	8.4929
N-acetylmethionine sulfoxide	3.6893	1.8834	3.37E-09	8.4729
butyrylcarnitine	0.31905	-1.6481	4.07E-09	8.3905
linoleoylcarnitine	4.3051	2.106	4.28E-09	8.3684
flavin adenine dinucleotide	2.0447	1.0319	4.28E-09	8.3684
5-oxoproline	2.04	1.0286	6.19E-09	8.2086
spermidine	0.30457	-1.7152	7.25E-09	8.1399
N-formylmethionine	2.1681	1.1165	8.68E-09	8.0617
fructose	0.25063	-1.9964	9.62E-09	8.0167
N-delta-acetylornithine	6.1407	2.6184	1.01E-08	7.9966
1-stearoyl-2-oleoyl-GPC 180/181	2.9262	1.549	1.08E-08	7.9657
myristoylcarnitine	2.5901	1.373	1.14E-08	7.9414
stachydrine	2.3057	1.2052	1.32E-08	7.879
prostaglandin E2	2.85	1.5109	1.36E-08	7.865
dimethylglycine	0.33703	-1.5691	1.61E-08	7.7938
glutarylcarnitine C5	6.7046	2.7451	1.77E-08	7.7511
dehydroascorbate	2.4321	1.2822	1.83E-08	7.737
thiamin diphosphate	2.9459	1.5587	1.84E-08	7.7354
6-phosphogluconate	0.28069	-1.8329	2.09E-08	7.6798
cysteine sulfinic acid	15.559	3.9597	2.32E-08	7.635
laurylcarnitine	4.5633	2.1901	2.45E-08	7.6116
2-aminoheptanoate	5.0048	2.3233	2.45E-08	7.6109
3-hydroxybutyrate	0.28464	-1.8128	2.76E-08	7.5595
galactonate	0.29535	-1.7595	3.04E-08	7.5165
heme	2.6974	1.4316	3.87E-08	7.4126
palmitoylcholine	4.7901	2.2601	6.58E-08	7.182
1-palmitoleoyl-3-oleoyl-glycerol 161/181	0.041627	-4.5864	9.57E-08	7.0191
proline	0.48182	-1.0534	1.04E-07	6.9841
glucose	0.2469	-2.018	1.19E-07	6.9253
Isobar fructose 1,6-diphosphate, glucose 1,6-diphosphate, myo-inositol 1,4 or 1,3-diphosphate	0.25113	-1.9935	1.27E-07	6.8952
*N*6-carboxymethyllysine	3.5522	1.8287	1.44E-07	6.8431
adenosine 2'-monophosphate	0.37507	-1.4148	1.53E-07	6.8144
γ-glutamylvaline	0.4289	-1.2213	1.66E-07	6.78
1,2-dilinoleoyl-GPC 182/182	0.48469	-1.0449	1.82E-07	6.739
cysteine-glutathione disulfide	6.6664	2.7369	2.08E-07	6.6817
sphingosine 1-phosphate	84510	16.367	2.28E-07	6.6424
inositol 1-phosphate	2.4492	1.2923	2.96E-07	6.5288
coenzyme A	2.8197	1.4956	4.77E-07	6.3217
methylmalonate	2.0726	1.0514	6.85E-07	6.1644
argininosuccinate	2.3416	1.2275	7.94E-07	6.1004

adenosine 5'-diphosphoribose ADP-ribose	296.84	8.2135	9.41E-07	6.0264
cytosine	0.40273	-1.3121	1.07E-06	5.9715
prostaglandin F2α	2.526	1.3369	1.33E-06	5.8747
1-arachidonoyl-GPI 204	4.5093	2.1729	1.42E-06	5.8478
2-aminoadipate	2.2999	1.2016	1.89E-06	5.7225
pyridoxal phosphate	2.563	1.3578	2.30E-06	5.6375
2-methylbutyrylcarnitine C5	765060	19.545	2.31E-06	5.6366
4-acetamidobutanoate	0.4209	-1.2485	2.34E-06	5.63
3-hydroxyisobutyrate	0.4529	-1.1427	1.07E-05	4.9726
1-1-enyl-oleoyl-GPE P-181	2.2525	1.1715	1.09E-05	4.9627
*N*4-acetylcytidine	0.25927	-1.9475	1.47E-05	4.8327
α-tocopherol	2.9592	1.5652	1.52E-05	4.8172
S-lactoylglutathione	7.2943	2.8668	1.64E-05	4.7841
1-oleoyl-GPG 181	17.495	4.1289	2.06E-05	4.686
3-hydroxybutyrylcarnitine 2	2.2086	1.1431	2.53E-05	4.5961
γ-glutamylhistidine	0.21373	-2.2261	2.94E-05	4.5314
N-stearoyltaurine	2.3196	1.2139	4.66E-05	4.3312
erucate 221n9	5.1422	2.3624	5.11E-05	4.2915
1-palmitoyl-GPS 160	0.2838	-1.817	5.49E-05	4.2603
solanidine	3.9245	1.9725	7.79E-05	4.1085
allo-threonine	2.8341	1.5029	8.31E-05	4.0806
1-1-enyl-stearoyl-GPE P-180	2.4186	1.2741	8.36E-05	4.0777
indolelactate	0.40907	-1.2896	0.00010553	3.9766
dihydroxyacetone phosphate	0.49472	-1.0153	0.00011992	3.9211
stearate 180	2.5486	1.3497	0.00013296	3.8763
guanosine 3'-monophosphate	9.9393	3.3131	0.00013297	3.8763
oleoyl ethanolamide	5.5757	2.4792	0.00013672	3.8642
imidazole propionate	5.7128	2.5142	0.00017238	3.7635
10-nonadecenoate 191n9	787210	19.586	0.00019433	3.7115
2-hydroxy-3-methylvalerate	3.4332	1.7795	0.00022024	3.6571
adenylosuccinate	0.4792	-1.0613	0.00039836	3.3997
adenosine 5'-diphosphate	5.684	2.5069	0.00043821	3.3583
eicosenoate 201	3.6288	1.8595	0.00098573	3.0062
linolenate α or γ 183n3 or 6	0.39434	-1.3425	0.0010827	2.9655
arachidonate 204n6	3.4764	1.7976	0.0011001	2.9585
adrenate 224n6	3.2377	1.695	0.0012023	2.92
oleate/vaccenate 181	2.8644	1.5183	0.0012045	2.9192
2'-deoxyadenosine	4.6742	2.2247	0.0017159	2.7655
3-indoxyl sulfate	5.7551	2.5248	0.0019744	2.7046
galactose 1-phosphate	0.41487	-1.2693	0.0020376	2.6909
fructose-6-phosphate	0.42295	-1.2415	0.0026612	2.5749
1-palmitoyl-GPG 160	2.135	1.0942	0.0026651	2.5743
docosahexaenoate 226n3	2.9339	1.5528	0.0049165	2.3083
2-oleoylglycerol 181	3.7374	1.902	0.0049592	2.3046
phenol sulfate	6.8315	2.7722	0.018664	1.729
docosadienoate 222n6	2.3406	1.2269	0.026193	1.5818
gluconate	0.48884	-1.0326	0.031631	1.4999

Supplementary Table S2: Forebrain metabolites significantly different between young adult (P60) and prepubertal/juvenile mice (P21). 14 metabolites were significantly higher in the forebrain of P60 mice compared to P21 mice and 29 metabolites significantly lower. Significant metabolites had a greater than two fold-change between the groups (P60 relative to P21) and an FDR-adjusted $P < 0.05$.

**Metabolite**	**Fold change (*P60 vs. P21*)**	**log2(FC)**	***p*.adj**	**-LOG10(p)**
N-methylnicotinic acid (trigonelline)	0.19463	-2.3612	3.67E-10	9.435
stachydrine	0.45015	-1.1515	4.19E-10	9.3774
4-guanidinobutanoate	0.4767	-1.0689	4.19E-10	9.3774
adenosine	2.3352	1.2235	8.42E-10	9.0749
2'-deoxycytidine	0.36112	-1.4694	1.30E-08	7.8863
2'-deoxyadenosine	3.5413	1.8243	7.61E-08	7.1189
sphingomyelin d182/230, d181/231, d171/241	2.7127	1.4398	1.39E-07	6.8575
palmitoylcholine	2.2075	1.1424	1.53E-07	6.8148
allantoin	0.46968	-1.0902	2.82E-07	6.5501
hypotaurine	0.49554	-1.0129	2.82E-07	6.5501
7-methylguanine	0.073047	-3.775	3.34E-07	6.4768
ergothioneine	2.2677	1.1812	8.73E-07	6.0592
dimethylglycine	0.23344	-2.0989	1.24E-06	5.9073
pro-hydroxy-pro	0.42465	-1.2356	1.24E-06	5.9073
2-aminoheptanoate	0.31808	-1.6525	1.24E-06	5.9067
adenosine 2'-monophosphate	0.30743	-1.7017	2.81E-06	5.5508
putrescine	0.23335	-2.0994	3.47E-06	5.4601
5-methyl-2'-deoxycytidine	0.29698	-1.7516	4.12E-06	5.3846
inosine 5'-monophosphate	0.38271	-1.3857	8.45E-06	5.0729
adenosine 5'-diphosphoribose	2.2571	1.1745	8.46E-06	5.0726
uridine 5'-monophosphate	0.42684	-1.2282	2.97E-05	4.5274
3-methylcytidine	0.3973	-1.3317	3.07E-05	4.5133
cysteinylglycine	2.2831	1.191	3.80E-05	4.4198
glutathione	2.1769	1.1223	9.05E-05	4.0433
2'-deoxyinosine	0.44955	-1.1534	9.14E-05	4.0389
4-hydroxy-nonenal-glutathione	0.48304	-1.0498	0.00016992	3.7698
adenylosuccinate	0.25492	-1.9719	0.00026016	3.5848
N4-acetylcytidine	0.21734	-2.202	0.000335	3.475
3-ureidopropionate	0.49395	-1.0176	0.00081704	3.0878
imidazole propionate	0.24348	-2.0382	0.00085564	3.0677
palmitoyl ethanolamide	2.5653	1.3591	0.00086244	3.0643
S-lactoylglutathione	2.2332	1.1591	0.00099882	3.0005
cytosine	0.22993	-2.1208	0.0010009	2.9996
guanosine 3'-monophosphate	0.20606	-2.2789	0.0011251	2.9488
urate	3.3978	1.7646	0.001443	2.8407
gamma-glutamylglycine	2.0527	1.0375	0.0016778	2.7753
2-hydroxy-3-methylvalerate	0.40993	-1.2865	0.0019479	2.7104
uridine 5'-diphosphate	0.3935	-1.3456	0.0020251	2.6936
phenol sulfate	3.3664	1.7512	0.0038704	2.4122
1-oleoyl-GPG 181	2.1815	1.1253	0.0046484	2.3327
indolelactate	0.30026	-1.7357	0.011497	1.9394
1-methylnicotinamide	0.42243	-1.2432	0.018371	1.7359
N-palmitoyltaurine	0.41276	-1.2766	0.02309	1.6366
